# Experiences and health-related quality of life following minimally invasive surgical repair for pectus excavatum – a mixed methods study

**DOI:** 10.1080/17482631.2026.2632366

**Published:** 2026-02-18

**Authors:** Louise Norlander, Agneta Anderzén-Carlsson, Mårten Vidlund, Ann-Sofie Sundqvist

**Affiliations:** a Department of Perioperative Medicine and Intensive Care (PMI), Karolinska University Hospital, Stockholm, Sweden; b Department of Cardiothoracic and Vascular Surgery, Faculty of Medicine and Health, Örebro University, Örebro, Sweden; c University Health Care Research Centre, Faculty of Medicine and Health, Örebro University, Örebro, Sweden

**Keywords:** Funnel chest, minimally invasive repair, mixed methods, Nuss, pectus excavatum, qualitative content analysis

## Abstract

**Background:**

Pectus excavatum is the most common congenital chest wall deformity. Minimally invasive repair for pectus excavatum (MIRPE) has demonstrated positive and psychsocial outcomes. The aim of this study was to gain a comprehensive understanding of health-related quality of life (HRQoL) in patients undergoing MIRPE.

**Methods:**

This prospective cohort study applied a modified mixed methods convergent design and included 19 individuals scheduled for MIRPE with follow-up 18 months postoperatively. The Nuss Questionnaire modified for Adults 10-item version (NQ-mA-10) and RAND-36 were administered pre- and postoperatively. Postoperative individual interviews explored participants’ lived experiences. Quantitative and qualitative data were integrated for a comprehensive analysis.

**Results:**

Significant improvements were observed in NQ-mA-10 total and subscale scores, whereas no significant changes were found in RAND-36 domains. Improvements in disease-specific HRQoL were supported by interview data. Integration of findings generated four themes: self-perceived exercise capacity, stronger sense of self, increased freedom in life and persistent chest-related issues.

**Discussion:**

Participants reported a stronger sense of self and greater freedom in life after MIRPE: However, persistent pain and fatigue highlight the need for further research and individualized postoperative care.

## Introduction

Pectus excavatum, or funnel chest, is the most common congenital chest wall deformity. The primary symptom of pectus excavatum is the depression of the sternum and the adjacent costal cartilages. Pectus excavatum is commonly asymmetrical with a rightward predominance (Fokin et al., 2009). The prevalence of pectus excavatum ranges from 1 in 250 to 1 in 400 people (Biavati et al., 2020; Rea & Sezen, 2020), and it is 3–5 times more common in men than in women (Fokin et al., 2009). The deformity can cause both psychosocial and physical symptoms. For some individuals, pectus excavatum is associated with chest pains, decreased exercise capacity, palpitations, dyspnoea and syncope (Nuss & Kelly, 2010). For other individuals, even a mild deformity can cause extended psychosocial distress with low self-esteem and withdrawal from social activities (Krille et al., 2012). Living with pectus excavatum can impose a heavy burden, affect mental well-being, place limitations upon life (Norlander et al., 2024), and negatively affect health-related quality of life (HRQoL) (Alaca & Yüksel, 2021).

HRQoL is defined as how well people function in their life and their perceived well-being in the physical, mental, and social domains of health (Hays & Reeve, 2008). This parameter forms the basis for this study. Traditionally, there are two main categories of HRQoL measurements: generic and disease-specific HRQoL. Generic measurements assess general HRQoL, despite eventual illness, and often are applicable for healthy individuals and focus on subjective well-being (Hays & Reeve, 2008). In contrast, disease-specific measurements assess HRQoL associated with a specific disease or condition and are capable of capturing more subtle effects associated with that specific situation (Fayers & Machin, 2016). When applicable, generic and disease-specific measurements can be combined to cover all aspects of HRQoL (Chen et al., 2005). Both generic and disease-specific measurements are operationalizations of individuals’ perceived HRQoL. Nevertheless, it is challenging to capture all the nuances of a patient’s experience due to the nature of self-reported outcome measurements, which most often have only predetermined questions and response options. Qualitative research methods are therefore necessary for exploring other aspects of HRQoL, as they enable more in-depth elaboration (Tonon, 2015).

Traditionally, treatment for pectus excavatum has been considered to be purely for cosmetic reasons, and surgical indications have been debated (Mohamed et al., 2023; Walsh et al., 2023). Today, minimally invasive surgical repair for pectus excavatum (MIRPE) is the gold standard of treatment for these individuals. During the procedure, one or more convex metal bars are placed beneath the sternum with thoracoscopic guidance, thereby pushing the chest wall depression outwards. The bars are secured and left in place for two to four years to allow the chest to maintain its new shape before being surgically removed (Nuss et al., 1998). MIRPE has previously shown positive results in terms of both the physical (Jaroszewski et al., 2022) and psychosocial well-being of HRQoL (Viggiano et al., 2022). However, no studies have described patients’ experiences with MIRPE during the postoperative period. Furthermore, no studies have compared qualitative data with self-reported HRQoL data to gain more in-depth knowledge of patients’ postoperative experiences. Therefore, the overall aim of this study was to gain a comprehensive understanding of participants’ HRQoL following MIRPE. The specific research questions were as follows.

1. How do individuals experience their well-being after MIRPE? (Qualitative)
2. Does HRQoL change after MIRPE, and if so, how? (Quantitative)
3. What patterns are identifiable when comparing the experiences of HRQoL and measured self-reported outcomes of HRQoL? (Mixed-methods)

## Methods

### Design and participants

**Figure 1.**
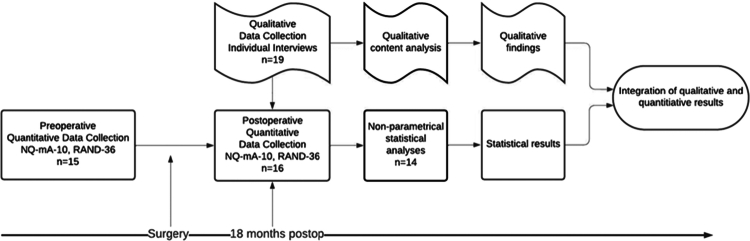
Flow chart of the study design.

This prospective cohort study applied a modified mixed methods convergent design (Creswell, 2017). The mixed methods convergent design refers to the simultaneous collection of quantitative and qualitative data, which are analysed separately and then merged. We made the following modifications: 1.) We used only postoperative interview data, as these data were rich in comparisons from the time before and after surgery. 2.) The quantitative data used were comparisons of pre- and postoperative HRQoL, as both sets of data are needed to describe any changes. The study design was based on a qualitative approach, and sample size was determined thereafter. This study was reported in accordance with the consolidated criteria for reporting qualitative research checklist (Tong et al., 2007). The study design is displayed in Figure 1.

Consecutive sampling was applied to enrol individuals who were scheduled for MIRPE at a university hospital, with a catchment area including the south and middle of Sweden. Eligible participants were first recruited by mail, followed by a telephone call. A total of 27 individuals were invited; of these, five declined to participate, and three had their surgeries postponed due to the COVID-19 pandemic and were excluded. Therefore, 19 participants were included in the study.

### Data collection and measurements

Data were collected between the 21^st^ of February, 2020, and the 5^th^ of October, 2023 at two time points: before MIRPE and 18 months after MIRPE.

#### Qualitative interviews

**Figure 2.**
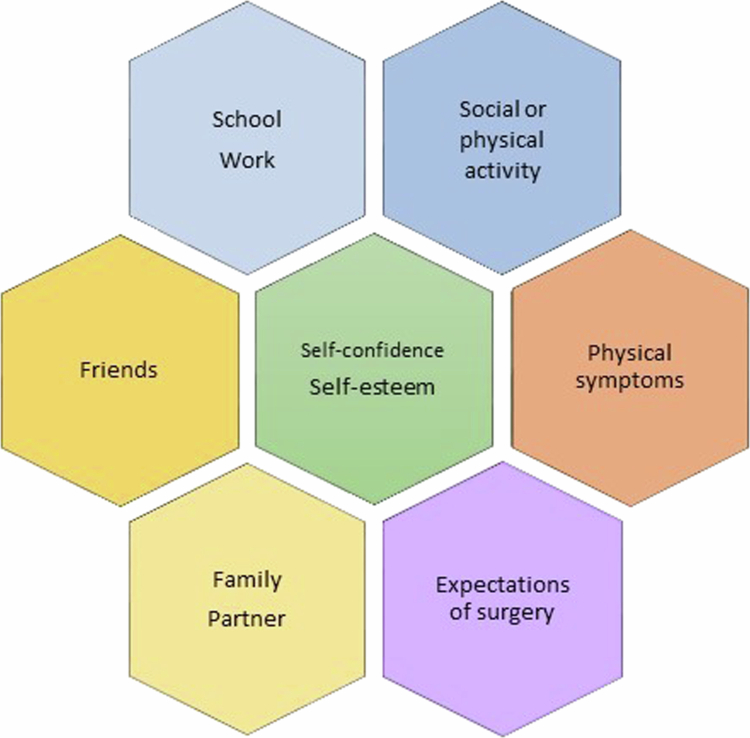
Interview topic guide (Norlander et al., 2024)

Qualitative individual interviews were conducted 18 months after MIRPE. Most of the interviews (n = 12) were conducted by the first author, and the remaining interviews were conducted by the second and last authors (n = 7). The interviews were semistructured. The opening question was as follows: “How can you describe your life after the surgery for your funnel chest?”. A topic guide (Figure 2) was used to ensure that the interviews covered important topics. The same guide was used in a previous study (Norlander et al., 2024), and the different topics in the guide have previously been described as central aspects of those living with a funnel chest (Roberts et al., 2003). The interviews were conducted by telephone because of the varied residential areas of the participants. The interviews lasted between 18 and 67 minutes, with a mean duration of 34 minutes. They were audio recorded and later transcribed verbatim by a professional transcriber.

#### HRQoL survey

Quantitative data were collected with web-based questionnaires consisting of the Nuss Questionnaire modified for Adults 10-item version (NQ-mA-10) (Norlander et al., 2021), the RAND-36 (Hays & Reeve, 2008) and a study-specific form for demographics.

The NQ-mA-10 is a 10-item disease-specific HRQoL questionnaire for individuals with pectus excavatum. The questionnaire is divided into two subscales: the psychosocial subscale (items 1–7) and the physical subscale (items 8–10). Each item is scored on a 4-point Likert scale, with the total score ranging between 10 and 40. Higher scores indicate better HRQoL. The NQ-mA-10 is a translated and culturally adapted Swedish version (Norlander et al., 2021) of the original NQ-mA (Krasopoulos et al., 2006). Although the instrument refers to measurements in adult patients, the original version was tested on individuals from the age of 14 and was therefore considered suitable for the current study.

The RAND-36 (Hays & Morales, 2001) is a 36-item generic HRQoL questionnaire that includes eight domains: physical functioning, role-physical, bodily pain, general health, vitality, social functioning, role-emotional, and mental health. Scores on each domain range from 0 to 100, with higher scores indicating better HRQoL. Additionally, participants completed a study-specific form that included questions regarding sex, age, height, weight, education and occupation. At the time of follow-up, participants were first approached by telephone and scheduled for a telephone interview; after the interview, participants received mail with a link to the web-based questionnaires.

### Data analysis

#### Qualitative data analysis

The qualitative data were analysed using content analysis with an inductive approach (Graneheim & Lundman, 2004). The transcribed interviews were controlled with the interviews recorded by the first author and edited for eventual misconceptions. The transcripts were then read thoroughly several times by the first author to obtain a sense of the whole. Meaning units (i.e., words or phrases) were identified, condensed, and labelled with a code. Codes were thereafter organized into categories based on similarities and differences in their manifest meanings. The analysis was an iterative process between all the authors until a consensus was reached. NVivo 14 software was used to manage the data.

#### Statistical analyses

Response options on the questionnaires were ordinally levelled, and thus, nonparametric statistical methods were used. However, the data for each subscale are expressed as percentages and means ± standard deviations so that the results can be compared with those of previous studies. Pre- and postoperative HRQoL scores were compared using the Wilcoxon signed rank test. Rosenthal’s *r* was calculated to estimate the effect size (ES) of the dependent group differences (Rosenthal & Rosenthal, 1991). The magnitude of *r* was categorized as follows: *r* > .1 = small, *r* > .3 = moderate, and *r* > .5 = large (Maher et al., 2013). The threshold for statistical significance was *P* < .05. Statistical analyses were performed via IBM SPSS Statistics version 25.0.

#### Integration of results

The results from the qualitative and quantitative analyses were merged to gain a more comprehensive understanding than could be derived from qualitative and quantitative results alone. The qualitative categories were compared with the outcome of specific HRQoL items. Each experience category was paired with NQ-mA-10 items with similar content and compared to determine whether they were confirmed, contradicted, or complemented one another. Based on the findings of this comparison, an abstract meaning of the merged results formed a theme. The integration is visually displayed in a side-by-side joint display (Fetters et al., 2013; Younas & Durante, 2023) in the results section.

### Ethical considerations

The study was planned and conducted in accordance with the Declaration of Helsinki (World Medical Association W, 2024) and approved by the Regional Research Ethics Board of Uppsala, Sweden (2018/365), and the Swedish Ethical Review Authority (2019-01177 and 2019-01177B). Written informed consent was obtained from all participants before the start of the study. For participants <15 years old, additional written informed consent was obtained from parents or guardians. Confidentiality was guaranteed for all participants and was especially considered in the use of quotations from the interviews, which were coded with numbers so that no participant could be identified.

## Results

The characteristics of the 19 participants are presented in Table I. All 19 participants participated in the interviews; however, two participants did not respond to any of the questionnaires. Of the remaining 17 participants, two were nonresponders at baseline, and one was lost to follow-up; thus, 14 participants were ultimately included in the pairwise statistical analyses.

**Table I.** Patient characteristics at baseline.

n	19
Male	17 (89.5)
Female	2 (10.5)
Age, mean (min–max)	22 (14–36)
Height (cm) mean (min–max)	183 (167–194)
Weight (kg) mean (min–max)	71 (52–105)
Highest education	
Ongoing compulsory	1 (5.3)
Compulsory	4 (21.0)
Upper secondary	9 (47.4)
University	1 (5.3)
Missing	4 (21.0)
Occupation	
Employed	8 (42.1)
Student	6 (31.6)
Job applicant	1 (5.3)
Missing	4 (21.0)

Data are given as n (%) unless otherwise noted.

### Interview results

The analysis of the interviews resulted in five categories describing the manifest content of the interviews. The categories were labelled as follows: “The painful initial time after surgery”; “I have gained increased physical capacity”; “I feel mentally stronger”; “I am less limited in my daily life”; and “It did not turn out as I imagined”. The findings showed that, overall, the participants experienced the initial time after surgery as difficult, with many participants experiencing pain and limitations in daily living. The first category covers the experiences of the first weeks and months after surgery, while the other categories reflect the overall experience from surgery to the 18-month follow-up. Hence, after that first difficult period, the participants gradually started to feel stronger both physically and mentally. They no longer experienced the physical limitations associated with the funnel chest, and simultaneously, they experienced increased self-esteem and confidence in their new looks of their chest. They also experienced fewer limitations in social situations and overall daily living. However, for some patients, the surgery did not turn out as they had hoped for. They experienced no changes at all or experienced adverse outcomes, including persistent pain, discomfort from the bars and other surgical complications still affecting their daily life. Below, the details of the categories are described.

#### The painful initial time after surgery

This category describes the first few weeks and months after surgery and how challenging this period was for the participants in many aspects. The participants most often mentioned the pain. Although they had been thoroughly informed about the pain they were most likely to experience after surgery, it was not possible to imagine to what extent and how it would interfere with daily living. Normal physical reactions such as laughing, coughing, or sneezing could be excruciating. One participant described it as *“If I were about to sneeze, I was genuinely afraid… a sneeze could make me lose my breath and then I had to rest for 5, 10, 15 minutes”* (Participant 8, 20 years). Only one participant reported that they experienced little pain during the first few days after surgery, after which they reported no pain.

The pain and fatigue many experienced after surgery made every task feel demanding, and the participants needed both physical and mental help from close individuals. Some participants expressed a decline in mental health during this period, and the COVID-19 pandemic exacerbated this effect. The participants felt isolated, as some expressed concerns of contracting COVID-19 and how that could interfere with their recovery. However, the pandemic also had a positive outcome, as it made remote work possible. Participants who otherwise would not have had any chance to go to work or school due to pain or fatigue could now engage from their home. Other individuals had physically demanding work and could not return to work for many months and needed extended sick leave and pain medication prescriptions due to persistent pain.

Although most of the participants initially expressed physical constraints, most of them described how they gradually improved over time. Despite the difficult initial time after surgery, most participants stated that the surgery was worth it: *“I would have done it again. I think it’s 100% worth it, it’s how do you say it... no pain, no gain”* (Participant 10, 17 years). Nevertheless, they emphasized that it was important to be aware that surgery is not a quick fix. However, some who had gone through surgery due to physical ailments said that they could not understand how anyone would go through it for cosmetic reasons because of the pain they experienced.

#### I have gained increased exercise capacity

The second category, “I have gained increased exercise capacity,” refers to the physical gains that the participants experienced after the surgery. For example, they no longer got out of breath as easily as before. The participants reported that their lungs now had more space for expansion and that it was easier for them to take deep breaths: *“It was so nice to get rid of it* [the pressure on the lungs]*. I no longer got short of breath from climbing stairs, which felt so good”* (Participant 3, 16 years). The participants also experienced increased exercise capacity; they described it as the heart receiving more space, which made cardio training especially easier. The breathlessness that some patients had experienced before surgery during physical activity was gone. Someone said that the most important change was not fainting anymore, whereas others stated that the increased exercise endurance was more important because exercising was more fun.

#### I feel mentally stronger

The surgery had a positive effect on the participants’ mental well-being. Before surgery, many participants had felt different, deviant and self-conscious about their chest every time they saw it. After the surgery, they reported feeling “normal” and no longer needed to think about it. They explained that they felt more comfortable in their body and were no longer conscious or ashamed when showing their chest to others. The participants described how their self-confidence and self-esteem had increased after surgery. It meant a lot to be like everyone else and to feel like one in the crowd. “*It feels better* [to be on the beach now]*, because you look more normal, like everyone else, I don’t have a hole anymore. That feels good.”* (Participant 17, 17 years).

#### I am less limited in my daily life

Not having to constantly think about their funnel chest made participants feel less limited in life. Before surgery, participants had avoided certain situations in which they were expected to remove their clothes, such as going to the beach or joining friends at parties or trips. After surgery, the participants instead reported that they could now join in such activities, as they no longer felt that they had a misshapen chest. It meant a lot to participants that their daily life had improved compared to before surgery*: “Of course it has meant a lot to me, to not have to think about this whole thing that 'Okay, in this situation it might be appropriate to take my shirt off, damn that’s hard' or so. Instead, now I can just enjoy the situation I’m in, make use of it and have a good time…”* (Participant 1, 29 years). One participant reported that the surgery had improved his relationship with his child and that they were now able to do things together as a family, which they had not previously experienced. Others described that they enjoy swimming and sunbathing, which were activities that individuals without a funnel chest took for granted.

The participants also reported that after the surgery, clothes fit better and that they did not have to think much about what clothes to wear. One woman described how bras and bathing suits had been difficult to find earlier because of her sternal depression, and she was now happy to finally have found a bikini that fit. Another participant described how shirts had just been hanging on his shoulders preoperatively, without any chest in between; after the surgery, his chest filled out the shirt, and he felt much more comfortable.

#### It did not turn out the way I imagined

Although many participants experienced a lot of positive changes after surgery, there were some aspects that did not turn out as expected. Some participants did not experience any changes after surgery at all; other participants were no longer able to do things they used to do before surgery; and some participants were unsure if they would have done the surgery at all if they were given the option again. The participants described sensing the bar on one or both sides of the chest. Some said they could not lay on that side where they were sore because of the chafing of the bar. They described that they were more careful than usual because of the bar, as they did not want to risk dislocating it. Some stated that it was reasonable to avoid certain activities, such as skiing and heavy lifting, until the bar was removed, yet others felt limited.

Persistent pain was also described as something that would come after long working days when being tired or exhausted or even when waking up in the morning after a night’s sleep. However, for some extensive pain interfered with daily living, resulting in the inability to work, sleep, or live their life as they used to do: *“Right now, it’s just worse than before in every way. I’m in pain every day, I can’t work out as hard, I can’t perform at work as hard, I can’t live as good, I exclude things. So, you can definitely say that… I have adjusted my life to my injuries…”* (Participant 7, 26 years). Other participants were overall disappointed with the long recovery period and reported that they had still not regained full strength after 18 months.

Some participants suffered from surgery-related complications. They reported infections, dislocations of the bar and reoperations, with prolonged hospital visits as a consequence. They also reported that support from health care was insufficient. They stated that they had not been informed about all possible complications, that health professionals did not pay attention to their concerns, and that they were not met with respect. While these participants did not generally advise against the surgery, they emphasized that others who consider surgery should make informed decisions with the full knowledge of the potential outcomes. One person regretted the surgery, but others reasoned differently: *“Based on the conclusion that I had known about the outcomes before I had the surgery, I probably wouldn’t have done it. The cons outnumber the pros. Sadly, that’s how it went. … But, I don’t regret doing it* [the surgery]*, because it was a great leap. … I’m proud I did it, but unfortunately, it did not turn out the way I imagined.”* (Participant 19, 31 years).

### Statistical analyses

There were no significant differences in scores on any domain of the RAND-36 between pre-MIRPE and post-MIRPE (Table II). The score on the social functioning domain increased after surgery, and the difference was almost significant. Disease-specific HRQoL, which was assessed with the NQ-mA-10, significantly differed between baseline and post-MIRPE across all domains (Table II). Compared with the total NQ-mA-10 score at baseline, the score at follow-up was significantly higher (*z* = 3.30, *N*—Ties = 14, *p* < 0.001), with a large ES. Both the psychosocial subscale and the physical subscale of the NQ-mA-10 showed a positive increase between baseline and post-MIRPE (*z* = 3.30, *p* < 0.001; *z* = 2.18, *p* = 0.03), both yielding large ES. The baseline and follow-up means, as well as the results of the Wilcoxon signed-rank test, are presented in Table II.

**Table II.** Total and subscale scores for the NQ-mA-10 and RAND-36 at baseline and follow-up.

	Baseline		Follow-up			
	Mean ± SD	Min–max	Mean ± SD	Min–max	*p*	ES
NQ-mA-10						
Total	27.3 ± 5.8	18–37	36.1 ± 4.9	25–40	<.001	.88
**Psychosocial**	18.5 ± 5	11–26	26.1 ± 2.8	19–28	<.001	.88
1. How chest looks without shirt	1.73		3.44		<.001	.9
2. Teasing because of chest looks?	3.87		3.87		1.0	0
3. Avoid doing things because of chest?	2.67		3.87		.003	.8
4. Hides chest?	2.4		3.62		.004	.76
5. Bothered because of how chest looks?	2.2		3.81		.002	.83
6. Feels shy/self-conscious because of chest?	2.6		3.81		.007	.72
7. Feels bad about oneself?	3.07		3.62		.033	.57
**Physical**	8.7 ± 2.3	4–12	10 ± 2.4	4–12	.029	.58
8. Has problem exercising?	3.13		3.44		.157	.38
9. Chest causes shortness of breath	2.4		2.94		.084	.46
10. Chest is the cause to be tired	3.2		3.62		.145	.39
**RAND-36**						
PF	89.3 ± 16.2	35–100	89.1 ± 20.6	35–100	.359	.25
RP	85.5 ± 30	0–100	85.9 ± 34.1	0–100	.891	.04
BP	84.8 ± 23.3	22.5–100	81.3 ± 25.3	10–100	.068	.49
GH	66.0 ± 25	15–100	73.4 ± 22.9	25–200	.146	.39
VT	63.1 ± 22.9	20–100	59.7 ± 24.3	5–95	.260	.3
SF	75.8 ± 26.9	25–100	84.4 ± 24.8	25–100	.054	.52
RE	73.3 ± 36.1	0–100	83.3 ± 23.3	0–100	.260	.30
MH	69.9 ± 18	32–92	73.3 ± 17.8	48–100	.160	.38

NQ-mA-10: Nuss questionnaire modified for adults 10-item version (Norlander et al., 2021), PF: physical functioning, RP: role-physical, BP: bodily pain, GH: general health, VT: vitality, SF: social functioning, RE: role-emotional, MH: mental health. Higher scores indicate better health-related quality of life. The significance level was determined as *p* < .05. ES: Effect size calculated with Rosenthal’s r, whereas >.1 = small, >.3 = moderate, and >.5 = large.

### Mixed-method integration

The qualitative and quantitative results were merged by comparing the results and examining differences and similarities to generate a meaningful understanding (Table III). The qualitative category “The painful initial time after surgery” was not paired with any of the items in the NQ-mA-10, as it relates to a period (first weeks and months after surgery) not measured at follow-up (18 months after surgery). Additionally, pain was not evaluated in the NQ-mA-10; thus, no item was suitable for comparison with this category.

**Table III.** Side-by-side joint display over qualitative, quantitative and mixed-methods integration.

Qualitative results	Quantitative results	Mixed methods integration
I have gained increased exercise capacity. *“If I ran 5 kilometres before surgery, I got really breathless, and a bit dizzy... […] But now when I run 7 kilometres, it is no problem, sure it’s hard to run, but it’s not anywhere near as hard as before, now I get tired in my legs instead of the lungs”* (Participant 18, 19 years)	Item 8, Has problem exercising (*p* = .157) Item 9, Chest causes shortness of breath (*p* = .084) Physical subscale (*p* = .029)	**Confirming**. *Increased self-perceived exercise capacity*
I feel mentally stronger. *“I’m actually not a withdrawn person. But the self-esteem has gotten so much better, I’m more secure in myself. Now, I have no problem joining my friends to the beach”* (Participant 11, 19 years)	Item 1, Satisfied with how chest looks (*p* < .001) Item 2, Teasing because of your chest looks (*p* = 1.0) Item 5, Bothered because of chest looks (*p* = .002) Item 6, Feels shy/self-conscious (*p* = .007) Item 7, Feels bad about oneself (*p* = .033)	**Confirming**. *Strengthened sense of self*
I am less limited in my daily life. *“Well, you can now come along for things without having to think twice about it. For example, if I were gonna go away skiing, I’d have to decline if you were gonna bath in the sauna or stuff like that. Then, I’d have to turn down the entire trip to avoid any situations where everybody is gonna bathe. … I would not* [turn down that kind of trips today]. *It’s so much fun, of course, that I can now come along for such things”* (Participant 5, 25 years)	Item 3, Avoid doing things because of chest (*p* = .003) Item 4, Hides chest (*p* = .004)	**Confirming**. *Increased freedom in life*
I did not turn out the way I imagined. *"My energy is still, or rather it's much worse than before* [the surgery]*, and my lung capacity has also gotten... a bit worse because I'm so out of shape after everything that's happened."* (Participant 14, 36 years)	Item 10, Chest is the cause to be tired (*p* = .135)	**Complementing**. *Persistent chest related issues*

The significance level was determined as *p* < .05.

#### Self-perceived exercise capacity

The qualitative category “I have gained increased exercise capacity” described the participants’ experience that physical activities were more easily performed 18 months after surgery than before surgery. When the content of the qualitative category was compared with the outcome of the specific NQ-mA-10 items 8 (Has problem exercising) and 9 (Chest causes shortness of breath), the results are contradictory, as no significant changes in these variables were identified after surgery. However, as the qualitative finding is in line with the overall scores on the total physical subscale of the NQ-mA-10, which indicated significantly better HRQoL after surgery (Table II), the results were considered confirming one another (Table III).

#### Strengthened sense of self

The category “I feel mentally stronger” included experiences of how participants were happier and now felt normal. This category also described an increase in self-esteem and confidence. The category was paired with the NQ-mA-10 items 1 (How chest looks without a shirt), 2 (Teasing because of chest looks), 5 (Bothered because of chest looks), 6 (Feels shy/self-conscious because of chest looks), and 7 (Feels bad about oneself), as well as the total psychosocial subscale. All paired items (except for item 2) as well as the total psychosocial subscale showed a significant increase in HRQoL after surgery (Table II), thus supporting the qualitative results (Table III).

#### Increased freedom in life

The fourth category, “I am less limited in my daily life,” refers to how participants felt less limited by the looks of their chest 18 months after surgery; they could now easily engage in both social and physical activities and not be bothered by their chest. The category was compared with NQ-mA-10 items 3 (Avoid doing things because of chest) and 4 (Hides chest), as well as the total psychosocial subscale. Items 2 and 3, as well as the total psychosocial subscale, showed significant increases in HRQoL after surgery (Table II), thus supporting the qualitative results (Table III).

#### Persistent chest-related issues

The final category, “It did not turn out the way I imagined,” described how a few participants did not experience the expected changes after surgery and how some suffered from diverse outcomes because of bar dislocation and infections, which caused them to be tired and unable to keep up with work or social life. The NQ-mA-10 item 10 (Chest is the cause of feeling tired) was paired with the category and showed no significant change after surgery; therefore, the results complement each other (Table III), as they provide different aspects of physical issues, where tiredness is one of them.

## Discussion

To our knowledge, this is the first study to integrate qualitative findings related to post-MIRPE experience with HRQoL data, thus providing a new dimension to the topic. The aim of this study was to gain a comprehensive understanding of participants' HRQoL experiences after MIRPE. The qualitative findings revealed that the period immediately following surgery is challenging and is characterized by extensive pain. However, after the first few weeks and months, most participants reported increased exercise capacity and felt stronger mentally. The participants also experienced fewer limitations in life due to their pectus excavatum. For some patients, however, the surgery did not meet their expectations.

Disease-specific HRQoL significantly improved after surgery on the total, psychosocial, and physical subscales of the NQ-mA-10. However, no statistically significant changes were observed in any of the RAND-36 domains. Key findings derived from the integration of qualitative and quantitative data identified four themes: *self-perceived exercise capacity, strengthened sense of self, increased sense of freedom*, and *persistent chest-related issues*.

The increased exercise capacity that participants experienced, including greater physical strength, improved exercise tolerance, and reduced breathing difficulties, aligns with the findings of previous studies. It is known that lung function improves after MIRPE, both at rest and during exercise and among both children (Liu et al., 2022) and adults (Jaroszewski et al., 2022). However, the effects of MIRPE on cardiopulmonary function are more controversial, although a relief of cardiac compression has been established (Raggio et al., 2024). Research has indicated that patients’ perceived change in exercise capacity is often greater than objective measures of pulmonary function or aerobic exercise tolerance (O'Keefe et al., 2013). This is similar to the results of our study, where participants’ descriptions of changes in exercise capacity were stronger than those captured by the NQ-mA-10. While HRQoL measurements often use predetermined questions with limited response options, these tools do not always allow participants to elaborate on their feelings or the personal value of MIRPE. Therefore, qualitative approaches may be beneficial for better understanding the individual impact of surgery on health outcomes, such as self-perceived exercise capacity.

The strengthened sense of self expressed by participants as feeling more “normal” with increased self-esteem and confidence echoes findings from previous qualitative studies. Roberts et al. (Roberts et al., 2003) noted that children who underwent MIRPE felt stronger and more confident, indicating greater personal empowerment. Similarly, Kim et al. (2011) reported that children experienced increased empowerment postsurgery. Moreover, the previous established improvements in both generic and disease-specific HRQoL (Mohamed et al., 2023) after MIRPE correspond to our findings to some extent. Although there were no significant improvements in the comparable domains of the RAND-36 after MIRPE, the HRQoL changes measured with the NQ-mA-10 confirm that participants' experiences with their sense of self are consistent.

The participants experienced increased freedom in life, partly because they were no longer avoiding activities due to concerns about how their chest looks. Preoperative avoidance typically involves social interactions (Norlander et al., 2024). Additionally, the RAND-36 domain social functioning showed a nonsignificant increase in social functioning after surgery. This mirrors previous findings, which indicate that children engage more in certain activities (Roberts et al., 2003) and experience a greater sense of social belonging after MIRPE (Kim et al., 2011). Social isolation can have profound effects on adolescents' brain and behavioural development, with lifelong consequences (Orben et al., 2020), thus making it critical to address this phenomenon. Our findings, together with those of previous studies, suggest that MIRPE holds significant value for improving social interactions.

However, for some participants, chest-related issues persisted after MIRPE. A few participants experienced persistent pain, which hindered their ability to resume normal activities 18 months after surgery. This finding highlights that pain remains a crucial factor to consider after surgery. Neither the NQ-mA-10 (Norlander et al., 2021) nor the original NQ-mA (Krasopoulos et al., 2006) includes a specific item assessing pain, suggesting that a complete understanding of HRQoL after MIRPE cannot be achieved solely through these disease-specific instruments. Pain was assessed via the RAND-36, and our results suggest that participants experienced more pain postsurgery, although this difference was not statistically significant. On the basis of these findings, incorporating pain assessments, either by adding pain-related items to existing instruments or by using a separate pain assessment tool for ongoing issues, may be valuable. This could help evaluate pain over time and potentially guide more personalized postoperative pain management strategies.

Although the small sample size of this study limits the generalizability of the findings, it is notable that the participants who experienced more postoperative complications were all older than 25 years. Higher age at surgery has been linked to more complicated repairs and a higher incidence of complications (Jaroszewski et al., 2016; Kim et al., 2005). Some participants in our study reported not being fully informed about all potential risks, including the increased risks associated with older age. The benefits and risks of MIRPE should be carefully considered on a case-by-case basis and clearly communicated to patients preoperatively so that they can make informed decisions about surgery.

Overall, our results suggest that the HRQoL data confirm and complement the experiences described by participants. Assessing HRQoL before and after MIRPE is both valuable and relevant, and the NQ-mA is the recommended instrument for this purpose (Dunning et al., 2024). However, our findings highlight discrepancies in reported and self-perceived exercise capacity, as well as the lack of pain assessment. Therefore, patient‒clinician interactions play a crucial role in understanding how patients experience the postoperative period, even 18 months after surgery. As previously described, an additional pain assessment tool could be beneficial for evaluating how patients are affected by complications. Future research should explore how pain and discomfort affect individuals both during the time the bars remain in place and after bar removal.

We recognize that this study has several limitations. First, we initially planned to include 20–25 participants, but data collection was halted early because of the COVID-19 pandemic. Nevertheless, the collected data are still considered sufficient in terms of heterogeneity, which highlighted for a diverse collection of experiences across genders and ages (Malterud et al., 2015). Therefore, the sample size is not believed to have affected the credibility the qualitative findings, although it limits the statistical power and generalizability of the quantitative results. Second, telephone interviews can be considered less advantageous in qualitative studies, as nonverbal communication, such as facial expressions and body language, is lacking; however, these interviews are valid and trustworthy alternatives to traditional face-to-face interviews (Saarijärvi & Bratt, 2021). Telephone interviews may be preferable to face-to-face interviews when discussing sensitive topics, as they provide interviewees with a greater sense of anonymity, potentially making it easier for them to share personal experiences (Sturges & Hanrahan, 2004).

The dependability of the study was considered during the data collection and analysis through thorough documentation of the research process (Ahmed, 2024). The mixed-method integration was visually demonstrated in the side-by-side joint display for more easily understandable interpretation, which is also recommended as one of the core quality criteria for mixed methods research (Hirose & Creswell, 2022). In the mixed methods integration, we choose to include only the statistical results of the NQ-mA-10. Owing to the small sample size, the RAND-36 results were considered exploratory and not suitable for the integration, as there was a substantial risk for type II errors (Akobeng, 2016). Additionally, when assessing HRQoL, disease-specific instruments capture aspects related to pectus excavatum that generic instruments cannot (Norlander et al., 2021). The results from the NQ-mA-10 measurements were therefore considered more appropriate for inclusion in the integration.

The confirmability of the study was possibly threatened, as the same author (LN) conducted the majority of the interviews, identified meaning units, and categorized codes into categories and subcategories during the analysis. However, all the authors controlled and confirmed the analyses, and the research group discussed the findings together, thereby enhancing the trustworthiness of the results. Furthermore, the participants in the study were all recruited from one high-volume MIRPE department. Compared with low-volume departments, high-volume departments performing MIRPE have reported fewer complications (Linton et al., 2022). This may have affected the participants’ experiences and led to more positive reports of HRQoL in this study, thus limiting its generalizability.

In conclusion, this study highlights the importance of integrating both qualitative and quantitative data to fully understand the impact of MIRPE on HRQoL. While significant improvements were described by patients and observed in disease-specific HRQoL, participants described self-perceived exercise capacity as suggesting additional benefits not fully captured by HRQoL instruments. Importantly, the lack of statistically significant changes in generic HRQoL should not be interpreted as absence of effect, but rather in light of the study´s limited statistical power. The study also emphasized the ongoing challenges some patients face, such as persistent pain, which is not adequately addressed in available HRQoL instruments. These findings underscore the value of personalized, patient–clinician interactions in assessing postoperative experiences and highlight the need for future studies with larger cohorts to enable age-stratified analyses and long-term follow-up after bar removal, including pain management and the impact on quality of life.

## Acknowledgements

We sincerely thank all individuals with funnel chest who generously shared their experiences with us. Your openness and willingness to participate made this research possible.

## Data Availability

The datasets generated and analysed during the current study are not publicly available because data sharing was not included in the ethical approval for this research. Participants did not provide consent for their data to be shared beyond the research team. Access to the data may be considered on a case-by-case basis, subject to additional ethics review and data protection regulations.

## Source(s) of support

LN was financed by grants from the Swedish state under the agreement between the Swedish government and the county councils, the ALF agreement (OLL-967445) https://www.researchweb.org/is/oll. LN was also financed by grants from the Örebro County Research Committee (OLL-941242) https://www.researchweb.org/is/oll. The funders had no role in study design, data collection and analysis, decision to publish, or preparation of the manuscript.
